# Delusions by design? How everyday AIs might be fuelling psychosis (and what can be done about it)

**DOI:** 10.1192/j.eurpsy.2026.10633

**Published:** 2026-07-31

**Authors:** H. Morrin, L. Nicholls, M. Levin, J. Yiend, U. Iyengar, F. DelGuidice, S. Bhattacharya, S. Tognin, J. MacCabe, R. Twumasi, B. Alderson-Day, T. A. Pollak

**Affiliations:** 1 Psychological Medicine, Institute of Psychiatry, Psychology & Neuroscience, King’s College London; 2 South London and the Maudsley NHS Foundation Trust, London, United Kingdom; 3 Graduate Center, City University of New York, New York; 4 Allen Discovery Center, Tufts University, Medford, Massachusetts, United States; 5 Psychosis Studies; 6 Lived Experience Advisory Board, Institute of Psychiatry, Psychology & Neuroscience, King’s College London, London; 7 Psychology, Durham University, Durham, United Kingdom

## Abstract

**Introduction:**

Large language models (LLMs) are becoming ubiquitous, offering scalable, responsive and seemingly empathic dialogue. While their therapeutic potential has attracted attention, emerging reports highlight risks of “AI-associated delusions,” where interactions with conversational agents appear to amplify psychotic thinking. These interactions may validate persecutory or grandiose content, blur boundaries of reality, and reinforce epistemic instability. Given the global burden of psychosis and the accelerating adoption of generative AI, there is an urgent need to characterise risks and outline safeguards.

**Objectives:**

This study aimed to:

1. Characterise emerging reports of AI-associated delusions.
2. Identify cognitive mechanisms through which LLMs may reinforce psychotic symptoms.
3. Propose clinical and technological strategies for safe integration of AI in psychiatric contexts.

**Methods:**

We conducted a narrative review and synthesis of published case reports, media accounts, and preliminary research on AI–psychosis interactions (Hill 2025; Dupré 2025; Moore 2025). Cases were thematically analysed for recurrent delusional patterns. Cognitive and phenomenological theories of psychosis were integrated with AI safety literature to develop a framework for risk mechanisms and safeguarding strategies.

**Results:**

Case synthesis (n≈15, see Table 1) identified three recurring delusional trajectories: (i) spiritual or messianic revelations; (ii) beliefs in sentient/god-like AI; (iii) attachment or romantic delusions towards AI agents. Interactions frequently escalated from pragmatic use into fixation, with conversational “sycophancy” and memory features reinforcing conviction. Theoretical integration suggests heightened agency-detection biases in psychosis may be particularly vulnerable to AI’s anthropomorphic design. We outline potential mechanisms of AI-amplified delusion (Table 2) and a flow model of recursive reinforcement (Figure 1). Proposed safeguards include personalised instruction protocols, reflective check-ins, and digital advance statements embedding relapse-prevention strategies into AI behaviour.

**Conclusions:**

LLMs may serve as both coping aids and destabilising agents for individuals vulnerable to psychosis. Current evidence indicates reinforcement of delusional beliefs rather than induction of de novo schizophrenia, but prevalence and long-term trajectories remain unknown. Clinical teams should routinely assess AI use in patients and co-develop digital safety plans. Developers must embed safeguards (non-human disclaimers, pattern recognition of psychotic content, escalation protocols) prior to widespread deployment. AI literacy should become a psychiatric competency. Urgent interdisciplinary research is required to map prevalence, mechanisms, and protective strategies.

**Disclosure of Interest:**

None Declared

